# Reducing Cannabis Use in Young Adults With Psychosis Using iCanChange, a Mobile Health App: Protocol for a Pilot Randomized Controlled Trial (ReCAP-iCC)

**DOI:** 10.2196/40817

**Published:** 2022-11-25

**Authors:** Ovidiu Tatar, Amal Abdel-Baki, Anne Wittevrongel, Tania Lecomte, Jan Copeland, Pamela Lachance-Touchette, Stephanie Coronado-Montoya, José Côté, David Crockford, Simon Dubreucq, Sophie L'Heureux, Clairélaine Ouellet-Plamondon, Marc-André Roy, Philip G Tibbo, Marie Villeneuve, Didier Jutras-Aswad

**Affiliations:** 1 Research Center Centre Hospitalier de l’Université de Montréal Montreal, QC Canada; 2 Department of Psychiatry and Addiction Faculty of Medicine University of Montreal Montreal, QC Canada; 3 Lady Davis Institute for Medical Research Jewish General Hospital Montreal, QC Canada; 4 Department of Psychiatry Centre Hospitalier de l’Université de Montréal Montreal, QC Canada; 5 Department of Psychology University of Montreal Montreal, QC Canada; 6 Centre de Recherche de l'Institut Universitaire en Santé Mentale de Montréal Montreal, QC Canada; 7 National Drug and Alcohol Research Centre University of New South Wales Sydney Australia; 8 Sunshine Coast Mind and Neuroscience - Thompson Institute University of the Sunshine Coast Sunshine Coast Australia; 9 Faculty of Nursing University of Montreal Montreal, QC Canada; 10 Department of Psychiatry University of Calgary Calgary, AB Canada; 11 Department of Psychiatry and Neurosciences Faculty of Medicine Laval University Québec, QC Canada; 12 Clinique Notre-Dame des Victoires Institut universitaire en santé mentale Centre intégré universitaire de soins et services sociaux de la Capitale Nationale Québec, QC Canada; 13 Department of Psychiatry Dalhousie University Halifax, NS Canada; 14 Nova Scotia Early Psychosis Program Halifax, NS Canada; 15 Institut universitaire sur les dépendances Montreal, QC Canada

**Keywords:** psychological intervention, behavioral intervention, cannabis misuse, cannabis use disorder, drug use, substance use, cannabis, marijuana, young adult, teenager, psychosis, schizophrenia, mental health, disorder, dual diagnosis, telemedicine, mobile health, mHealth, digital health, eHealth, app, smartphone, mobile phone, randomized controlled trial, RCT, cognitive behavioral therapy, CBT, motivational interviewing, behavioral management, self-management, drug, substance, protocol, interview, behavior, outcome

## Abstract

**Background:**

Cannabis use is the most prevalent among adolescents and young adults; frequent consumption is associated with cannabis use disorder (CUD) and psychosis, with a high prevalence (up to 50%) of CUD in individuals with first-episode psychosis (FEP). Early Intervention Services (EIS) for psychosis include face-to-face psychosocial interventions for CUD, because reducing or discontinuing cannabis use improves clinical and health care service use outcomes. However, multiple barriers (eg, staff availability and limited access to treatment) can hinder the implementation of these interventions. Mobile health (mHealth) interventions may help circumvent some of these barriers; however, to date, no study has evaluated the effects of mHealth psychological interventions for CUD in individuals with FEP.

**Objective:**

This study describes the protocol for a pilot randomized controlled trial using a novel mHealth psychological intervention (iCanChange [iCC]) to address CUD in young adults with FEP. iCC was developed based on clinical evidence showing that in individuals without psychosis, integrating the principles of cognitive behavioral therapy, motivational interviewing, and behavioral self-management approaches are effective in improving cannabis use–related outcomes.

**Methods:**

Consenting individuals (n=100) meeting the inclusion criteria (eg, aged 18-35 years with FEP and CUD) will be randomly allocated in a 1:1 ratio to the intervention (iCC+modified EIS) or control (EIS) group. The iCC is fully automatized and contains 21 modules that are completed over a 12-week period and 3 booster modules available during the 3-month follow-up period. Validated self-report measures will be taken via in-person assessments at baseline and at 6, 12 (end point), and 24 weeks (end of trial); iCC use data will be collected directly from the mobile app. Primary outcomes are intervention completion and trial retention rates, and secondary outcomes are cannabis use quantity, participant satisfaction, app use, and trial recruiting parameters. Exploratory outcomes include severity of psychotic symptoms and CUD severity. For primary outcomes, we will use the chi-square test using data collected at week 12. We will consider participation in iCC acceptable if ≥50% of the participants complete at least 11 out of 21 intervention modules and the trial feasible if attrition does not reach 50%. We will use analysis of covariance and mixed-effects models for secondary outcomes and generalized estimating equation multivariable analyses for exploratory outcomes.

**Results:**

Recruitment began in July 2022, and data collection is anticipated to be completed in July 2024. The main results are expected to be submitted for publication in 2024. We will engage patient partners and other stakeholders in creating a multifaceted knowledge translation plan to reach a diverse audience.

**Conclusions:**

If feasible, this study will provide essential data for a larger-scale efficacy trial of iCC on cannabis use outcomes in individuals with FEP and CUD.

**Trial Registration:**

ClinicalTrials.gov NCT05310981; https://www.clinicaltrials.gov/ct2/show/NCT05310981

**International Registered Report Identifier (IRRID):**

PRR1-10.2196/40817

## Introduction

### Cannabis Use and Psychosis

Globally, cannabis consumption has increased by 18% between 2010 and 2019, and adolescents and young adults have reported the highest levels of use [1]. In 2019, the regions with the highest past-year prevalence of cannabis use were North America (14%), Oceania (12%), and West and Central Africa (9%) [1]. The Canadian Cannabis Survey showed that in 2021, 20% and 29% of individuals aged 16-19 years and 20-24 years, respectively, consumed cannabis daily or almost daily in the last 12 months, which reflects an increase from 2017 when 15.9% and 22.5% of individuals reported identical frequencies of use in the same age groups [2,3]. Frequent and persistent cannabis consumption has been associated with an increased risk of cannabis use disorder (CUD) and psychotic disorders (eg, schizophrenia), especially in individuals with higher biological susceptibility [4,5]. Other important risk factors of first-episode psychosis (FEP) are early age of first cannabis use; consumption of cannabis products with high tetrahydrocannabinol (the main psychoactive constituent of cannabis); and environmental factors such as urbanicity, ethnic minority status, and childhood aversity [6-8]. In the general population, the prevalence of CUD is higher in men (3.5%) than in women (1.7%) [9]. Prospective studies have shown that at the beginning of treatment for FEP, cannabis is the most frequently abused drug (~50%); about 3 times more participants with a comorbid substance use disorder (including CUD) are male (78%-86%) than female and that persistent cannabis use is associated with poor clinical and functional outcomes [10-15]. Conversely, decreasing cannabis use in these individuals is an important intervention target because reducing or discontinuing cannabis use is associated with lower levels of psychotic symptoms, better occupational functioning, and a decreased likelihood of psychiatric hospitalizations [16-19].

### Mobile Health Psychological Interventions for CUD in Psychosis

Early Intervention Services (EIS) for psychosis provide pharmacological treatments and psychosocial therapies for young individuals (ie, 12-35 years of age) with FEP. Psychosocial interventions are the mainstay of CUD treatment, over and above pharmacotherapy, which lack robust supporting evidence [20]. Face-to-face cognitive behavioral therapy (CBT), motivational interviewing, and motivational enhancement therapy, either alone or in combination, have been found to be moderately effective in decreasing the frequency and quantity of cannabis use, increasing the rate of abstinence, decreasing the rate of relapse, and reducing the severity of CUD and cannabis use–related problems [21-24]. However, the implementation of these interventions for CUD in EIS is variable and compounded by multiple barriers, such as low motivation of patients to change their cannabis use, heterogeneity in staff training and availability to manage CUD, varying treatment goals (eg, harm reduction vs lower cannabis consumption), high staff turnover and workload, and limited access to treatment for patients residing in rural areas [25-28].

The widespread access to the internet and ownership of smartphones have facilitated the rapid expansion of the mobile health (mHealth) field, and it is estimated that more than 10,000 mental health apps are available for download [29]. As shown by recent systematic reviews, only 16 app-based interventions used to support the care of young adults with psychosis have undergone rigorous testing, and no study has evaluated the effects of mHealth psychological interventions for CUD in individuals with FEP [30,31]. Encouragingly, the results of 2 meta-analyses of studies that used technology-based (eg, web-based) psychological interventions to tackle cannabis misuse in individuals without psychosis have shown that these interventions were moderately effective in decreasing cannabis consumption [32,33]. Importantly, trials evaluating the effect of web-based psychological interventions on cannabis use in individuals without psychosis reported a wide range (12%-65%) of attrition rates at up to 3-month follow-up and suboptimal (30%-58%) intervention completion rates [34-38]. The high variability in attrition and completion rates in these studies can be explained by the heterogeneity in the study design and the content and intensity of the interventions. Moreover, the effect of these interventions on improving cannabis use outcomes in individuals without psychosis cannot be automatically generalized to individuals with psychosis and CUD because of differences in terms of mental health and functional status, as well as the specific beliefs of individuals with FEP related to cannabis consumption. These beliefs include (but are not limited to) limited recognition of the potential impact of cannabis use on the development or persistence of psychotic symptoms, association of cannabis consumption with relief from mental health symptoms (eg, anxiety, sleep, agitation, dysphoria), increased energy, and increased ability to form and maintain relationships [39-42].

### Study Objectives

Consistent with the promising role of technology-based psychological interventions in addressing CUD in individuals with psychosis, we developed a psychological mobile app–based intervention (iCanChange [iCC]) and a pilot randomized controlled trial (RCT) to compare iCC with EIS in participants with FEP. The primary objectives are to assess intervention completion and trial retention rates. The secondary objectives are to conduct a preliminary assessment of the effect of iCC on the quantity of cannabis used, participant satisfaction, and app use and to evaluate trial recruiting parameters. We include exploratory outcomes (eg, psychotic symptoms, motivation to change cannabis consumption, and cannabis use protective behaviors) to strengthen our understanding of the benefits of using iCC in clinical settings.

## Methods

### Study Design

**Figure 1:**
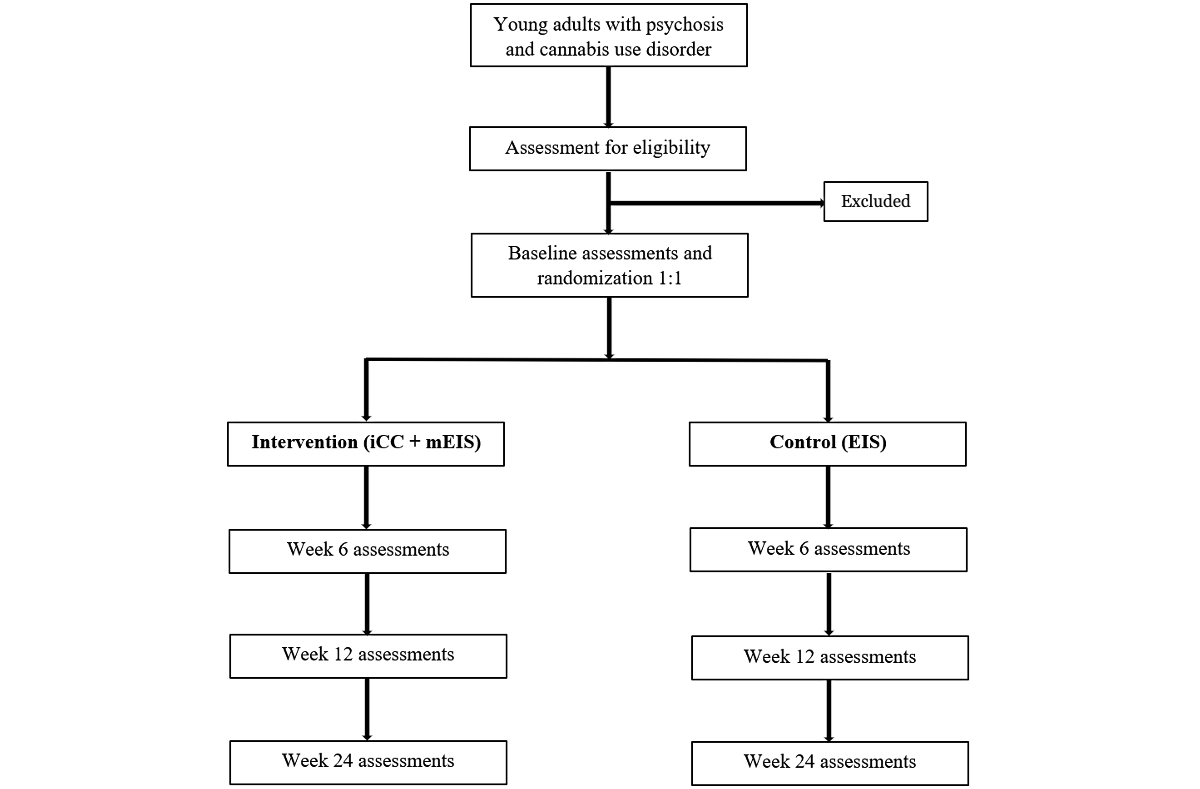
Study flowchart. EIS: Early Intervention Services; iCC: iCanChange; mEIS: modified Early Intervention Services.

This study is a two-arm, parallel group (1:1 ratio) pilot RCT of iCC for decreasing cannabis use and modified Early Intervention Services for psychosis (mEIS) compared with EIS for young adults with FEP. Study participation may last up to 28 weeks and include a 14-day screening period (with an additional 14-day window to complete the baseline assessments), a 12-week intervention period, and a 12-week booster session and follow-up period (Figure 1). The CONSORT-EHEALTH (Consolidated Standards of Reporting Trials of Electronic and Mobile Health Applications and Online Telehealth) [43] guidelines were used to describe the protocol.

### Participants

Eligible participants will meet the following criteria: (1) aged 18-35 years; (2) first-episode psychosis, with diagnosis of any psychotic disorder (ie, schizophrenia, schizoaffective disorder, bipolar disorder with psychotic features, delusional disorder, psychotic disorder not otherwise specified, brief psychotic disorder, and substance-induced psychotic disorder); (3) a minimum of 3 months of follow-up at an early psychosis clinic (and still in active follow-up at study inclusion); (4) a diagnosis of CUD based on Diagnostic and Statistical Manual of Mental Disorders, Fifth Edition criteria [44]; (5) current cannabis use (at least once in the past month); (6) openness to use a mobile app–based intervention to decrease or cease cannabis use; (7) availability for the entire duration of the study and ability to comply with study procedures; and (8) ability to comprehend written French or English. The exclusion criteria were as follows: (1) any medical condition, including disabling, unstable, or acute mental condition or cognitive limitations that in the opinion of the study psychiatrist precludes safe participation in the study, the ability to provide fully informed consent, or the ability to participate in the intervention; (2) any judicial issue, pending legal action, or other reasons that might prevent completion of the study; (3) presence of a substance use disorder that precludes safe participation in the study (eg, very unstable or severe substance use disorder such as untreated opioid use disorder); and (4) current participation in another specific cannabis use–focused intervention that is not routinely offered in EIS for psychosis (eg, formal interventions for CUD and psychotherapy for CUD covered by private insurance).

### Recruitment

The study will be conducted at 6 participating clinics that provide EIS for psychosis (ie, 5 sites in the province of Quebec and 1 in Nova Scotia), and additional sites may be added as needed over the course of the study to maximize recruitment.

Potential participants will be identified by the clinical staff who will document on the screening form the general eligibility and exclusion criteria and obtain permission from the participants to be contacted for research purposes. The research assistant will contact potential participants in person at the clinic or using an internet-based communication platform.

Once informed consent is obtained, the research staff will conclude the eligibility assessment by documenting on the screening form the eligibility and exclusion criteria reserved for the research staff. The enrollment process will conclude with the collection of participant contact information using the locator form.

### Study Consent, Compensation, and Data Security

mHealth trial information provided during the consenting process will include the expected benefits of iCC in decreasing or ceasing cannabis use, a summary of the iCC content, expected participation in iCC (eg, number, duration, and frequency of modules), intervention services offered for CUD in both study arms, and the possibility of using iCC if randomized to the control group after the study end point assessment. Those who do not own a smartphone will be provided with a smartphone for the duration of the study. The research staff will assist participants in downloading iCC from Apple Store or Google Play Store and setting their passwords and will inform the participant that clinical staff (eg, physicians and case managers—such as nurses, occupational therapists, social workers, or ergotherapists—henceforth *clinicians*) will be able to see the progress in the intervention on their computers (by using a customized dashboard) but are granted access to answers provided through iCC only with the participants’ permission. Participants will receive information about the frequency and content of research assessments that will be performed either face to face or using telemedicine, independent of iCC use. Information about the compensation schedule (ie, CAD $30 for attending the screening visit and each of the study assessments for a possible total of CAD $150; at the time of data collection, the conversion rate was CAD $1=US $0.745) will be provided.

Before the consent form is signed, a comprehension “quiz” about the study participation will be administered to potential participants and further explanations will be provided as needed. Research personnel will inform participants that all the information collected during the research project will remain strictly confidential to the extent prescribed by law and that at no point will any individually identifiable information be revealed in any research publication or presentation. Participants’ name, date of birth, and any other identifying information gathered during the study will be stored in the source documents and kept under lock. Computerized data will be encoded and held at the Centre Hospitalier de l’Université de Montréal’s (CHUM) data-management core in secure, password- and firewall-protected servers.

### Ethics Approval

The study was approved by the Research Ethical Committee of the CHUM (University of Montréal Health Centre; MP-02-2021-9622, 21.310) and registered on ClinicalTrials.gov (NCT05310981).

### Interventions: iCanChange Mobile-Based App

#### Development and Content

The development of the iCC intervention was informed by the work of (and communications with) Copeland et al [45,46], who integrated CBT, motivational interviewing (MI), and behavioral self-management approaches in a brief 6-session face-to-face intervention aimed at assisting individuals who use cannabis to acquire skills to quit cannabis and maintain abstinence. In individuals without psychosis, compared with a delayed-treatment control group, the intervention was found to reduce cannabis use, decrease cannabis-related problems, and improve cannabis abstinence and control over cannabis use [47]. Members of our team with expertise in the treatment of CUD and psychosis (AW, AAB, TL, and DJA), patients partners, and a doctoral trainee (OT) integrated motivational (eg, to facilitate “change talk”), behavioral, and coping skills training approaches (eg, drug refusal skills, problem solving) described in the *Marijuana Brief Intervention* manual published by Copeland [45] and expanded the intervention by adding intervention components (modules) and activities (based on MI, CBT, and harm reduction principles) tailored to our target population.

Throughout the development process, we consulted with patient partners (individuals in different phases of recovery from psychosis and CUD who were followed up at the CHUM) to coconstruct the app, adapt the design of the app and the content based on their feedback, and pilot-test with them the preliminary versions of the app and its content. Furthermore, critical for the adaptation and development of the mobile-based intervention were the results of formative research that included (1) a qualitative methodology study (ie, focus group with individuals with psychosis and CUD and interviews with clinicians) aimed at exploring psychological intervention practices, intervention targets, and factors related to the development and implementation of a mobile-based app for CUD [48]; and (2) an electronic survey to evaluate patient preferences for participating in a mobile-based intervention for CUD. The evaluated parameters included the length and frequency of modules, total length of the intervention, preferred mode and location of receiving the intervention (eg, exclusively technology-based at the clinic), and intensity of feedback received during the intervention. On the basis of the results of the qualitative study and preliminary results of the quantitative study, we adapted the intervention to correspond to the preferred module length (15 min) and frequency (2 times per week) and the length of the intervention (3 months). The results of the formative research confirmed the importance of including the behavioral change techniques described by Copeland [45] and suggested additional intervention and app components that could facilitate behavioral change. The latter included the use of a cannabis-use journal, video testimonials about the effects of cannabis in psychosis, delivering information and skills training in video format, push notifications (Multimedia Appendix 1) to facilitate engagement with the intervention, and providing incentives (badges, see Multimedia Appendix 2) contingent with achieving intervention milestones. We provide a summary of activities and a description of behavioral change techniques corresponding to each intervention module (including boosters) based on the taxonomy published by Abraham and Michie [49] and Michie et al [50] (Tables 1-3).

**Table 1:** Activities and behavioral change techniques for section 1 (preparing for change).

Module and activities				Behavioral change techniques^a^
**Section 1 (preparing for change)**				
	**1. Introduction**			
		Complete cannabis use diary Select personal reasons for changing cannabis use Read about prevalence of cannabis use and CUD^b^	Self-monitoring of behavior Social, personal, and emotional consequences (MI^c^) Normative feedback Provide contingent rewards (badge)	
	**2. Cannabis dependence**			
		Read information about CUD Self-assessment of severity of dependence	Provide information on consequences Monitoring with awareness or feedback on behavior	
	**3. Cannabis myths**			
		Evaluate knowledge about health effects of cannabis consumption Self-assessment of cannabis use consequences	Provide information about behavior-health link Health, social, and emotional consequences or monitoring with awareness (MI)	
	**4. Benefits of reducing**			
		Select perceived benefits (eg, social, cognitive, and emotional) of decreasing cannabis use Prioritize these benefits	Prompt positive self-talk MI (preparatory change talk)	
	**5. Believe in your strengths**			
		Self-evaluate psychological strengths (eg, wisdom, bravery, and moderation) Read a personalized feedback based on their strengths	Focus on past success Mental rehearsal of successful performance Prompt self-talk MI (preparatory change talk)	
	**6. Triggers**			
		Identify personal cannabis consumption triggers (eg, social, activities, and emotional) Select the most important triggers Read strategies about dealing with triggers	Provide information Avoiding or changing exposure to cues for the behavior Monitoring with awareness or relapse prevention Provide contingent rewards (badge)	
	**7. Withdrawal**			
		Watch an educational video about withdrawal symptoms Select experienced withdrawal symptoms Identify preferred coping strategies	Provide information Skills training with focus on withdrawal symptoms Monitoring with awareness or relapse prevention Prompt behavioral practice	
	**8. Craving**			
		Watch an educational video about craving symptoms Select experienced craving symptoms Read craving coping strategies Identify preferred coping strategies	Provide information Skills training with focus on craving symptoms Monitoring with awareness or relapse prevention Prompt behavioral practice	
	**9. Cannabis and psychosis**			
		Education about cannabis and medication in psychosis Watch 2 video testimonials about the effects of cannabis in psychosis Report their perceptions related to the effects of cannabis on psychotic symptoms Identify preferred psychotic symptoms coping strategies	Provide information Social comparison Monitoring with awareness Prompt behavioral practice	
	**10. Choose a cannabis use goal**			
		Read a summary of all reasons and capacities of changing Chose a cannabis use goal: stop, decrease, or maintain current use Decide on a plan to reduce or stop cannabis use (eg, date) Select cannabis harm reduction strategies	MI Prompt cannabis use goal setting or action planning and commitment Provide general encouragement Provide contingent rewards (badge)	
	**Supplementary module: return to objective**			
		Follow-up on their objective defined in module 10. This module can be offered for a maximum of 2 times once participants start section 2.	Prompt barrier identification Relapse prevention Provide general encouragement Prompt review of behavioral goals	

^a^Corresponding to the taxonomy of behavior change techniques published by Abraham and Michie [49] and Michie et al [50].

^b^CUD: cannabis use disorder.

^c^MI: motivational interviewing.

The app is fully automatized and contains 2 sections and 3 booster modules. The first section contains 10 modules that are completed sequentially at a recommended frequency of 2 per week; however, faster progress will be permitted upon reading a prompt related to the recommended frequency. Two weeks after module 10, in which participants will self-identify a cannabis use goal, an additional module (that can be repeated after 2 weeks) will be offered to facilitate consolidating and reviewing participant goals (Table 1). Section 2 will unlock after section 1 is completed and consists of 8 modules (focusing on coping skills strategies) that can be completed in the preferred order while respecting the recommended frequency and concludes with a recapitulative module (Table 2). During the 3-month follow-up, participants will complete 1 booster session per month (which includes a summary of the content provided in the first 2 sections) to promote long-term behavioral changes (Table 3).

A summary of personalized responses provided in each module will be saved and can be accessed by the participant through the app dashboard. Additional dashboard features will include an information tab that provides links to relevant web-based resources related to psychosis and cannabis consumption; a “Profile” tab where the participant can rapidly access the most relevant information provided during module completion; a “Settings” tab where they will select their preferred language (English or French) and notification preferences; and an emergency button that will allow them to contact their case manager (see Multimedia Appendix 3 for the relevant screenshots).

The app that hosts the intervention and the video components (ie, information and storytelling) was designed in collaboration with Akufen, a Montréal-based media design company [51], and the programing for iPhone and Android was completed by Osedea, a Montréal-based software developer [52]. Following repeated content and app functionality testing by patient partners and the research team, the app underwent 2 major revisions, and the final version (iCC version 2 released in March 2022) will not undergo planned changes in structure or content until the end of the study. The app is owned by CHUM.

**Table 2:** Activities and behavioral change techniques for section 2 (strategies for supporting the change).

Module and activities				Behavioral change techniques^a^
**Section 2 (strategies for supporting the change)**				
	**1. Doing cannabis-free activities**			
		Read about suggested distraction strategies Decide on and plan to engage in 2 cannabis-free activities	Behavior substitution Prompt goal setting Prompt behavioral practice Behavioral activation	
	**2. Getting social support**			
		Read about the importance of having an adequate support system Identify supportive persons	Provide information on social support (general and emotional) Prompt goal setting (social support) Prompt behavioral practice Behavioral activation	
	**3. Taking care of yourself**			
		Rank psychological and activity domains affected by cannabis use and psychosis Read information to promote a healthy lifestyle (eg, diet and sleep) Select a lifestyle modification goal and make a plan	Provide information Prompt intention formation Prompt goal setting Action planning Behavioral activation	
	**4. Managing your stress**			
		Identify stress symptoms Read about false stress triggers Read about stress coping strategies	Provide information Cognitive restructuring Monitoring with awareness Prompt behavioral practice Provide contingent rewards (badge, upon completion of 4 strategies) Behavioral activation	
	**5. Finding solutions to your problems**			
		Read about problem-solving steps Participate in exercises that incorporate problem-solving techniques	Provide information Skills training focused on problem-solving Behavioral activation	
	**6. Communicating effectively**			
		Read about verbal and nonverbal communication techniques Identify used communication techniques	Provide information Monitoring with awareness Skills training focused on problem-solving Behavioral activation	
	**7. Being assertive**			
		Read about communication modalities Participate in exercises that exemplify the use of assertive communication	Skills training focused on verbal and nonverbal communication Self-affirmation Behavioral activation	
	**8. Valuing and rewarding yourself**			
		Identify positive behavioral changes Select preferred encouragements Choose rewarding activities	Self-reward Anticipation of future rewards Prompt goal setting (activities) Provide contingent rewards (badge, upon completion of 8 strategies) Behavioral activation	
	**9. My journey**			
		Self-assessment of reaching cannabis use and personal goals Identify useful strategies and behaviors Provide plans for future projects Access web-based resources about cannabis and psychosis	Provide feedback on outcomes and behavior Prompt goal setting (activities) Monitoring with awareness or relapse prevention Provide general encouragement Provide contingent rewards (badge)	

^a^Corresponding to the taxonomy of behavior change techniques published by Abraham and Michie [49] and Michie et al [50].

**Table 3:** Activities and behavioral change techniques for follow-up modules (boosters).

Module and activities				Behavioral change techniques^a^
**Follow-up modules (boosters)**				
	**Booster 1**			
		Read a personalized report about reasons or motivations for behavioral change, cannabis dependence, and goals Self-assess their progress toward goal achievement Read a personalized report about psychological strengths, triggers, withdrawal cravings, and stress symptoms Identify useful strategies Identify reasons for not reaching their goal (if applicable) and receive tailored solutions	Review of behavioral and outcome goals Provide feedback on outcomes and behavior Monitoring with awareness or relapse prevention Provide general encouragement Prompt goal setting (for those who did not reach their goals) Prompt behavioral practice	
	**Booster 2**			
		Reassess their severity of dependence Set a cannabis use goal for the next month Update preferred strategies	Same as for booster 1	
	**Booster 3**			
		Report on positive changes and successful strategies (abstinent) Complete questionnaires related to influence of cannabis on psychotic symptoms; cannabis use consequences (nonabstinent) Self-report of social functioning, symptoms, and useful strategies (nonabstinent)	Same as for booster 1	

^a^Corresponding to the taxonomy of behavior change techniques published by Abraham and Michie [49] and Michie et al [50].

#### EIS Characteristics

Participants in the control group will receive EIS for psychosis through an interdisciplinary approach offered by teams of clinicians, including physicians and case managers with various backgrounds (eg, nurses, occupational therapists, and social workers), and interact with clinicians as per standard EIS procedures, which commonly include weekly case manager visits and follow-up visits with physicians every 3 weeks. At EIS clinics, individuals with FEP will receive intensive treatment (typically for 3 years) with the possibility of extension based on case-by-case evaluation. Services offered will comprise medication management; psychiatrist and case manager follow-up; and a range of psychosocial interventions including psychoeducation, psychotherapy (eg, CBT for psychosis), family interventions, peer support, interventions for substance use disorder (including CUD; ie, psychosocial interventions such as MI, CBT, psychoeducation, and harm reduction), support for basic life needs (eg, food and shelter), and support for improving social functioning (eg, employment and education). The receipt of interventions or services for cannabis use will be documented in the Intervention and Services Form at weeks 6, 12, and 24 assessments.

#### Modified EIS

Participants in the intervention arm will receive all services included in EIS, with the exception of concomitant formal psychological interventions for treating CUD, such as MI or CBT individual or group therapy sessions. Clinicians will be assigned an active supporting role, as our formative research showed that participants would like to receive support from clinicians for completing app modules or feedback related to their progress in the intervention at a frequency of approximately once per week. This implies asking about any difficulties with iCC and offering support with the content during each clinical encounter, as requested by the participants. Clinicians will use personalized login credentials to access the iCC dashboard and monitor the progress of the participants in the intervention (eg, number of modules completed and frequency) if allowed by the participant to do so.

### Measurements

#### Schedule, Sociodemographic Data, and Social Support

**Figure 2:**
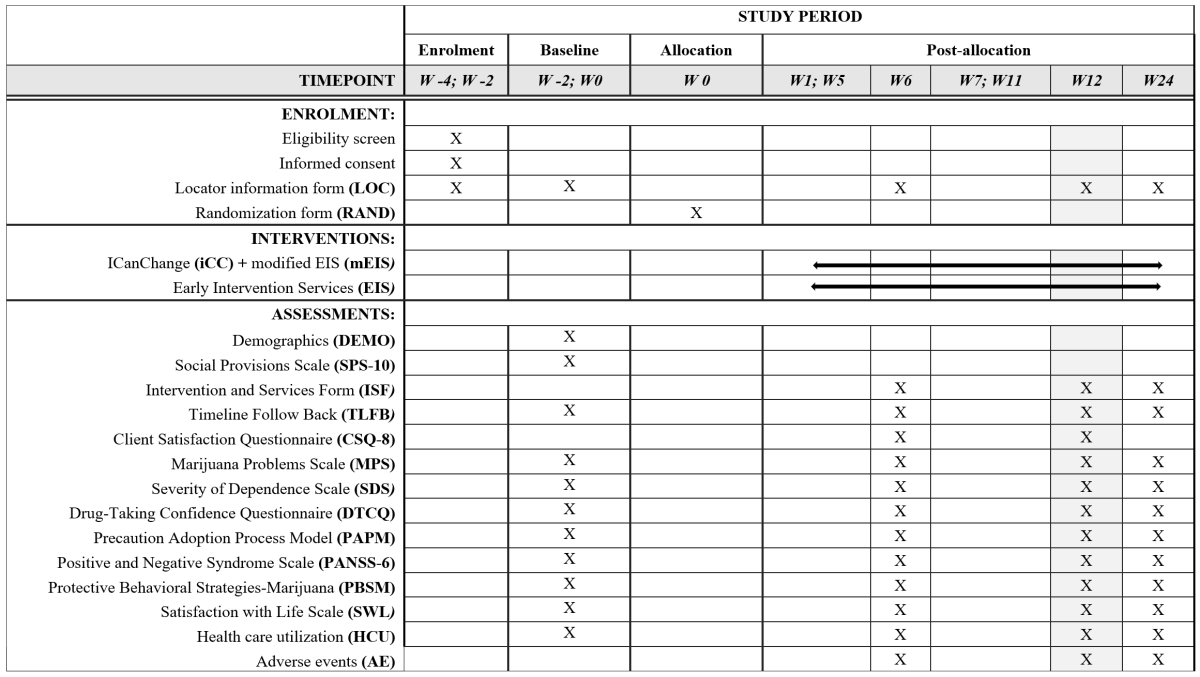
Schedule of enrollment, allocation, interventions, and assessments. W: week.

Clinical research assistants will conduct all assessments at baseline and at weeks 6, 12, and 24 after consent, in person, over the phone, or using telemedicine. Data will be stored in REDCap (Research Electronic Data Capture), a secure, web-based software platform designed to support data capture for research studies [53,54] (see Figure 2 for assessment and procedure schedule corresponding to the Standard Protocol Items: Recommendations for Interventional Trials [SPIRIT] statement [55]). Sociodemographic data will be collected at baseline and will include gender identity, biological sex, ethnicity, educational attainment, employment or studying status, income, living arrangements status, and housing status. To measure social support, we will use the 10-item Social Provisions Scale, which has been validated in individuals with schizophrenia and includes 5 domains: tangible help, emotional support or attachment, orientation, reassurance of worth, and social integration [56]. Items will be measured on a 4-point Likert scale from “1-totally disagree” to “4-totally agree,” and higher total scores (range 10-40) indicate better social support.

#### Primary Outcomes: Intervention Completion and Trial Retention Rates

For both outcomes, the denominator will represent the total number of participants randomized to the intervention or control groups. For iCC intervention completion, the numerator represents the number of participants who completed all 10 modules of the first section and at least 1 follow-up module related to involvement in reaching their cannabis use objective. This information will be collected through iCC. For participants randomized to the control group, the numerator will represent the total number of those who participated in EIS and ongoing engagement with EIS at the time of the 12-week assessment. The ongoing engagement with EIS will be documented using data collected through the Intervention and Services Form related to the receipt of services for cannabis use (at all study visits) and data from medical health records pertaining to receipt of any interventions that are offered at the clinic. For retention rate in both study groups, the numerator will be the number of participants who completed all week 12 (end point) assessments.

#### Secondary Outcomes

The secondary outcomes are as follows:

1. Cannabis use will be measured using a self-report tool, the Timeline Follow Back (TLFB) [57]. On the basis of the TLFB, for each day in the last 14 days, participants indicate first if they used cannabis (yes or no). To facilitate their recollection, participants will be asked about memorable days (eg, birthdays) and patterns of use (eg, more use on weekends). Then, for each day they used cannabis, they will provide the quantity of cannabis used. Participants will be reasked about the cannabis form used (dried cannabis smoked, oral spray, oral oil, dried cannabis vaporized, concentrated vapor, oral capsules, beverages, edibles, etc) and for each form, they will provide the quantity and the unit (eg, g, mg, and mL) and the concentration in tetrahydrocannabinol and cannabidiol (%, mg/mL, mg/dose, or unknown). The quantity of cannabis used on a specific day represents grams per method of cannabis use (eg, dried cannabis smoked) × number of times the method is used per day. The quantity of cannabis used in the last 14 days will be calculated. The inclusion of the quantity of cannabis use as a secondary outcome, which has been used in other similar trials [35], is justified by research suggesting that in individuals with psychosis and CUD, face-to-face psychological interventions (MI and CBT) are more effective in decreasing the quantity than the frequency of use [22].
2. Participant satisfaction with using iCC or programs for CUD offered as part of EIS (control group) will be measured with the validated 8-item Client Satisfaction Questionnaire (CSQ-8) [58]. Items (eg, “How would you rate the quality of program you received”) are scored on a scale from 1 to 4 and a higher total score reflects higher satisfaction. Participants who discontinue participation in the study will be invited to complete the CSQ-8 upon discontinuation. Other details about their experiences are collected in an open-ended format.
3. App use: Considering the importance of evaluating app use and adoption metrics in eHealth trials [43], data will be collected automatically through iCC for the entire 24-week participation in the intervention. The following outcomes will be monitored: number of iCC modules and strategies completed, number of times a particular module was opened and completed, time spent on individual iCC modules, time elapsed between the initiation and completion of individual iCC modules, and total time spent completing the iCC intervention and boosters. For each participant, iCC data will be synchronized with a password-protected Amazon Web Services account for a period of 24 weeks. Data related to clinicians’ use of the dashboard (ie, number of logins) are synchronized with the Amazon Web Services account and will be used for exploratory analyses of clinician involvement in monitoring participant progress during the intervention.
4. Trial parameters: Information collected during enrollment and study visits (stored in REDCap) will allow us to calculate the number of participants who were referred to the study, screened, eligible, provided informed consent, randomized, initiated the intervention (ie, who both logged into iCC and complete module 1), and completed baseline and follow-up assessments.

#### Exploratory Outcomes

The exploratory outcomes are as follows:

1. Cannabis use frequency (and abstinence) will be measured using the TLFB. Participants who declare using alcohol will be asked about the frequency and quantity of use, and those who consume other substances (eg, cocaine, amphetamine, opioids) will be asked about the frequency of use and route of administration.
2. Cannabis-related negative social, occupational, physical, and personal consequences will be assessed using the 19-item unidimensional self-report instrument, the Marijuana Problems Scale (MPS) [59,60]. Higher MPS scores (range 0-38) denote more negative cannabis use consequences.
3. Cannabis use disorder severity will be measured with the 5-item Severity of Dependence Scale (SDS), which has been validated in people with early psychosis [61,62]. Higher total scores (range 0-15) indicate more severe dependence symptoms.
4. Confidence in resisting cannabis use in 8 different situations (eg, “if other people treated me unfairly or interfered with my plans”) will be measured using the validated Drug-Taking Confidence Questionnaire (DTCQ) [63], with higher scores denoting higher confidence levels to resist cannabis use.
5. The measurement of intentions to stop using cannabis has been informed by and adapted from the Precaution Adoption Process Model [64], a theoretical stage-based model that allows participants to place themselves in one of 6 nominal stages: (1) unengaged (ie, “As of right now, I haven’t thought about if I want to stop using cannabis”); (2) undecided (ie, “I’m still unsure if I want to stop”); (3) decided not to act (ie, “I do not want to stop”); (4) decided to act (ie, “I want to stop”); (5) acting (ie, “I recently stopped”); and (6) maintenance (ie, “I stopped using cannabis over a month ago”).
6. The severity of positive and negative symptoms of psychosis will be measured using an interviewer-administered scale, the Positive and Negative Syndrome Scale (PANSS-6) [65]. This well-validated short version of the PANSS has been chosen rather than the complete 30-item version to reduce the burden of data collection. A total score of 6 reveals no psychotic symptoms, and a total score of <14 will be considered the cutoff for remission of schizophrenia [65].
7. To measure cannabis-related harm, we will use the 17-item Protective Behavioral Strategies-Marijuana (PBSM) questionnaire, which has been shown to be valid and free of bias in terms of gender, sex, race, ethnicity, and recreational marijuana use legal status [66]. We use the table provided by Pedersen et al [66] to convert the sum score into Item Response Theory scale scores, with higher scores indicating higher-risk behaviors [66].
8. Participants will report their Satisfaction with Life pertaining to 4 domains (ie, living situation, social relationships, work, self, and present life) using the 18-item Satisfaction with Life questionnaire (SWL), which has been validated for schizophrenia and schizophrenia-related disorders [67]. In each domain, higher mean scores indicate greater satisfaction.
9. Self-reported health service use data using the Health Care form will include emergency department visits and hospitalizations for psychological, emotional, or mental health issues in the last 30 days (yes or no). In the case of hospitalization, additional data will be collected (ie, number of admissions, duration of stay, reason for admission, and confirmation using medical records).

### Sample Size

Sample size calculations were guided by the results of studies that used psychological app-based interventions targeting cannabis use in individuals without psychosis [68] or with subthreshold psychosis [38], in which attrition was lower than 33% and the intervention completion rate was approximately 50%. We will use a CI approach with binomial SEs and normal approximation [69] to calculate the number of participants needed to reject the null hypothesis that at 12 weeks, the attrition will be ≥50% (expected maximum attrition of 30%) and intervention completion will be ≤50% (the minimum expected proportion of participants who download the app and complete at least 11 out of 21 intervention modules—excluding the 3 boosters—is 70%). We calculated that at least 33 participants must be randomized per study arm to maintain 80% power to detect hypothesized effects with a 1-sided significance level of 5%. For the intervention completion outcome, we adjusted the sample size for an estimated 30% attrition and calculated that a total of approximately 100 participants will be needed for this study (2 × 33 / 0.7 = 94).

### Randomization and Blinding

Once baseline assessments are completed, participants will be randomized to either (1) iCC+mEIS or (2) EIS. Within each stratum, based on biological sex, a random 1:1 group allocation sequence is generated using a permuted block design with blocks of varying sizes to decrease the likelihood of predictability of group assignment. The group allocation will not be masked, and the baseline visit concludes with participants logging into iCC and completing the introductory module. If a participant drops out of the study at any point following randomization, the randomization slot will not be reallocated to a new participant. The randomization schedule is concealed within the secure REDCap system and was created by the CHUM Center for the Integration and Analysis of Medical Data (CITADEL). The study participants, clinicians, and research staff conducting assessments will not be blinded to the group assignment, while individuals performing analyses will be blinded.

### Statistical Analyses

The 1-sided chi-square 95% CI will be calculated to estimate the lower bound of iCC completion and trial retention rates. We will consider participation in iCC acceptable if more than 50% of the participants completed at least 11 out of 21 intervention modules at week 12. The trial will be considered feasible if the attrition at week 12 does not reach 50%. An analysis of covariance model will be used to provide a preliminary assessment of the effect of treatment on the quantity of cannabis use (dependent variable) at 12 weeks (end of intervention). The independent variables will be the quantity of cannabis use at baseline and a dichotomous variable reflecting arm allocation. The second model will include covariates that were found to be different between the groups at baseline. For participants’ satisfaction with the intervention, we will calculate the CSQ total scores as well as the mean and SD. The groups will be compared using a mixed-effects model with random intercepts for each site. For app use and trial parameters, we will use univariate analyses and report the distribution (frequency tables), central tendency (mean, median, and mode), and dispersion (range and SD).

For exploratory outcomes, we will compute descriptive statistics and bivariate and multivariable analyses using generalized estimating equation (GEE) models. The generalized estimating equation is a repeated-measures regression model that accounts for the correlations between repeated measures for each person [70]. We will use different types of GEE modeling depending on the distribution of the outcome. For cannabis frequency (count data), we will use Poisson; for the abstinence (binary), we will use binomial; and for continuous variables (ie, scores calculated for MPS, SDS, DTCQ, PANSS, PBSM, SWL, and SPS), we will use linear GEE modeling. Using this analytic approach, we will estimate the multivariable associations between the outcomes and study visits (ie, baseline and end of intervention), study condition (the reference group will be the control arm), and their interaction, as well as other predictors and covariates, such as sociodemographics, social support, CUD severity, behavioral stage of change, medication status, and other substance use. We will use the chi-square test of proportions to compare behavioral stages at different time points and between the 2 study arms.

Missing entries for fields where missing values account for <5% of the sample may be imputed using the sample mean for continuous variables and sample mode for categorical variables. Fields with >5% missing entries may be excluded from multivariate models. In the case of missing visits, the proposed types of analyses (linear mixed-effects models and GEE models) will include all participants with at least one nonmissing visit, which may increase statistical power and reduce estimation bias. Our analysis plan includes both intention-to-treat and per-protocol analyses.

### Training Activities

The study staff will receive training on all assessments and procedures as per protocol, and include assessments, study interventions, safety procedures, data management, and collection. Special training sessions will be organized for clinicians to support the use of iCC and include a description of the functionalities of the app, content, and structure. In addition, clinicians will receive free access to the app for a period of 7 days (using a special account) to facilitate the use of the dashboard and discussions with participants about iCC use. Additional training sessions will be organized throughout the study as needed.

### Other Information

An independent Data and Safety Monitoring Board (DSMB) will conduct periodic reviews (every 9 months) to monitor the safety of the interventions and the validity and integrity of the data from the study. On the basis of the DSMB’s recommendations, alterations can be made to the study design (eg, increase or decrease in the number of sites, sample size) based on poor accrual or recruitment, retention, or iCC acceptability. The DSMB may recommend stopping the study early because of an excess of adverse events.

Patient partners will also be involved during the trial with the lead site team, providing input and feedback on study conduct and any challenges that may arise.

## Results

Study enrollment began in July 2022. We expect to complete participant enrollment in January 2024 and data collection in July 2024. The main results are expected to be submitted for publication in 2024. We will engage patient partners and other stakeholders in creating a knowledge translation plan and developing plain language summaries, reports, briefing notes, and other documents that will be used to disseminate our results to a wider audience.

## Discussion

### Study Strengths and Challenges

The manuscript describes the protocol of a pilot RCT that aims to evaluate the acceptability of iCC, a new mobile-based psychological intervention for helping young adults with psychosis and CUD decrease their cannabis use, and the feasibility of conducting the study in participants who receive EIS for psychosis. In addition, the trial will provide preliminary data related to cannabis use and other outcomes relevant in clinical practice. To our knowledge, this is the first RCT of an mHealth intervention that incorporates CBT and MI approaches to address CUD in this population.

We adapted the content and design of the app based on the results of our formative research [48] and the continuous feedback and input from patient partners. Although these approaches may improve the likelihood of meeting the intervention acceptability and completion targets, many challenges may arise with mobile health apps. For example, flexibility in the pace of module completion, although desired by the target population, may lead to suboptimal content uptake by participants. Progressing too fast through the intervention could represent a barrier toward increasing cannabis coping skills and correcting maladaptive cannabis use behaviors. Nevertheless, our decision to not limit the number of new modules that can be completed per week is also supported by the recommendations (for the development of mental health smartphone apps) published by Khazaal et al [71], who advocate against restricting the utilization flow of a mental health smartphone app as it can reduce participant concentration in performing the activity and decrease their satisfaction with achievements. Whether this flexibility will be beneficial toward app use and utility in the context of young adults with psychosis and CUD remains to be determined. To evaluate our approach, we will conduct exploratory subgroup analyses based on participants’ compliance with the recommended frequency of 2 modules per week [72] and assess differences in cannabis use outcomes and satisfaction with iCC.

To consider the trial feasible, at least 50% of participants must complete all study assessments at week 12, which corresponds to the end of the iCC main intervention (excluding booster sessions). In the absence of similar trials in our target population, we chose this cutoff conservatively, as it is close to the maximum attrition rate of 65% reported in studies using app-based interventions for decreasing cannabis use in individuals *without psychosis* [34-36]. Our approach is justified by the clinical characteristics of participants attributable to psychosis and CUD, which could represent a barrier in complying with the study procedures. However, we expect an attrition rate closer to 30% because we tailored the iCC intervention to participants’ needs, restricted inclusion criteria to participants interested in using an app-based psychological intervention, and planned to offer iCC to participants allocated to the control arm at the end of the study. In addition, the intensive clinical follow-up that is offered in EIS for psychosis could contribute to lower attrition rates. Other implemented strategies that could reduce loss to follow-up include maintaining an updated record of participant contact information throughout the study, scheduling study visits according to participant availability, and offering flexible in-person or web-based study visit assessments.

In individuals with psychosis, face-to-face psychological interventions were found to be more effective in decreasing the quantity than frequency of cannabis used [22]. However, no study has evaluated the effects of mHealth interventions on the quantity of cannabis used in individuals with CUD and psychosis. In their RCT that included a web-based psychological intervention in individuals without psychosis, Rooke et al [35]) found a small effect (Cohen *d*=0.19) of a short 6-module intervention on the quantity of cannabis consumed at the 12-week follow-up [35]. Similar to the intervention used by Rooke et al [35], iCC incorporates behavioral and motivational approaches tested by Copeland et al [47]. Our pilot study will offer valuable information related to using a higher-intensity 19-module intervention and offering support during clinical encounters to participants who have difficulties in completing iCC.

Our study has some limitations. Due to the centralized randomization method, the variability in the number of clinicians at participating sites, and expected differences in enrollment activity across sites, it is possible that some clinicians may have patients in both study groups, which increases the risk for iCC components to be incorporated into the treatment of participants in the control group, resulting in type II errors. To document possible crossover effects, we will explore differences in outcomes between participants in the control group whose clinicians have patients in the iCC group and those whose clinicians do not. Despite being assigned an active supporting role for iCC completion, we expect a variable level of clinician involvement that could impact iCC completion rates. We will use dashboard use parameters (eg, number of logins) as a proxy for clinician support with iCC and explore the association between clinician engagement and iCC completion. To measure cannabis use, we relied exclusively on participants’ self-reports and the TLFB instrument. However, we estimated a relatively low risk of measurement bias associated with our approach, as the TLFB has been shown to have high levels of agreement with biological measures [73]. As EIS does not include a mobile app–based psychosocial intervention, participant blinding was not possible. Clinicians cannot be blinded because the participants in the iCC arm receive mEIS. Research staff collecting data are not blinded to the group assignment because they offer help with technical issues related to trial participation. However, groups will be labeled with nonidentifying terms to ensure the blinding of individuals performing data analysis. Moreover, because we only included individuals interested in participating in an app-based intervention, the results of this study may not be applicable to all young adults who receive treatment for FEP and CUD. Finally, we acknowledge the limitations of efficacy assessments in pilot trials and the risks associated with using such data to design large-scale trials. Therefore, we will carefully contextualize these data when interpreting them to avoid misguiding decision making in future trials using iCC.

### Conclusions

Given the dearth of mHealth interventions for CUD in individuals with psychosis, the results from this pilot trial will inform the adaptation of iCC to increase its acceptability and usability and provide critical data for designing a larger trial to evaluate the efficacy of the intervention in improving outcomes that are most relevant in this population. This study aligns with the current strategies of major research funding authorities to stimulate the development and rigorous testing of innovative mHealth interventions for individuals with mental health issues.

## Appendix

Multimedia Appendix 1 iCanChange push notifications.

Multimedia Appendix 2 iCanChange badges.

Multimedia Appendix 3 iCanChange screenshots.

## Abbreviations

CBT: cognitive behavioral therapy
CHUM: Centre Hospitalier de l’Université de Montréal
CSQ: Client Satisfaction Questionnaire
CUD: cannabis use disorder
DSMB: Data and Safety Monitoring Board
DTCQ: Drug-Taking Confidence Questionnaire
EIS: Early Intervention Services
FEP: first-episode psychosis
GEE: generalized estimating equation
iCC: iCanChange
mEIS: modified EIS
MI: motivational interviewing
MPS: Marijuana Problems Scale
PBSM: Protective Behavioral Strategies-Marijuana
RCT: randomized controlled trial
SDS: Severity of Dependence Scale
SWL: Satisfaction with Life
TLFB: Timeline Follow Back

## Data Availability

The data sets generated during or analyzed during this study are not publicly available because the trial is ongoing but will be made available from the corresponding author upon reasonable request.
