# Supplementary material for: Reducing Cannabis Use in Young Adults With Psychosis Using iCanChange, a Mobile Health App: Protocol for a Pilot Randomized Controlled Trial (ReCAP-iCC)
Source: JMIR Res Protoc. 2022 Nov 25;11(11):e40817. doi: 10.2196/40817 (PMC9736767; doi:10.2196/40817)
Supplement: Multimedia Appendix 1 [file resprot_v11i11e40817_app1.pdf]

Appendix 1. Push notification schedule iCC

Advice corresponds to screens that appear in the application during the intervention but are not linked to push notifications.

Reminders correspond to push notifications. Each reminder has the same title: Hello [first name]

Type	Time	Condition	Content
**Section 1**			
Reminder	1 day after the first journal entry (in the introduction)	Only if the first module was not started OR completed	The introduction module is available
Reminder	1 day after module 1 was started	Only if module 1 was not completed	The introduction module is available. You can continue!
Reminder	3 days after the last module was completed	Only if module X was not started or completed	The module on "Cannabis Dependence" is waiting for you<br>The module on "Cannabis Myths" is waiting for you<br>The module on "Benefits of Reducing" is waiting for you<br>The module on "Believe in Your Strengths" is waiting for you<br>The module on "Triggers" is waiting for you<br>The module on "Withdrawal" is waiting for you<br>The module on "Craving" is waiting for you<br>The module on "Cannabis and Psychosis" is waiting for you<br>The module on "Choose a Cannabis Use Goal" is waiting for you
Reminder	1 day after module X was started	Only if module X was not completed	Don't forget to finish your module
Advice	Anytime during the intervention period (sections 1 and 2)	If less that 2 days have elapsed between modules	You are fast! For iCanChange to be more effective, it is recommended to wait 2 days between modules or strategies in order to take the time to integrate and practice the new info.
Advice	Anytime during the intervention period (sections 1 and 2)	If more than 3 modules are completed on the same day	Wow, you're really fast! There's no rush… Take the time to practice the new modules or strategies.
Reminder	1 week after completing section 1		You can redo all the modules in Section 1
Reminder		If more than 3 days have elapsed from the last journal entry	Don't forget to use your journal to keep track of your cannabis use
Reminder	Every 8th day	Only if the journal is not completed for the previous 7 days	Writing in your journal can help you better understand your cannabis use

**Section 2**			
Reminder	3 days after completing module 10 in section 1		Did you start working toward your goal?
Reminder	4 days after completing module 10 in section 1	Only if no strategy has been started or completed	Applying a strategy could help you on a day-to-day basis
Reminder	6 days after completing module 10 in section 1	N/A	Experiencing withdrawal symptoms? Redoing the withdrawal module could help
Reminder	8 days after completing module 10 in section 1	N/A	Are you having cravings? Redoing the craving module could help.
Reminder	2 weeks after completing module 10 from section 1 and 4 weeks after completing module 10	N/A	How is your cannabis use goal coming along? Good, we hope!
Reminder	1 week after completing strategy "Doing cannabis-free activities"	N/A	Have you done cannabis-free activities?
Reminder	1 week after completing strategy "Getting social support"	N/A	Don't forget that there are people around you who can help.
Reminder	1 week after completing strategy "Taking care of yourself"	N/A	How is your healthy mind in a healthy body goal coming along?
Reminder	1 week after completing strategy "Managing your stress"	N/A	You can manage your stress. It's time to relax.
Reminder	1 week after completing the strategy "Finding solutions to your problems"	N/A	Every problem has a solution. Take time to find it
Reminder	1 week after completing the strategy "Communicating effectively"	N/A	Practicing new communication strategies helps
Reminder	1 week after completing the strategy "Being assertive"	N/A	Don't hesitate to say what you're thinking and to assert yourself
Reminder	1 week after completing the strategy "Valuing and rewarding yourself"	N/A	Don't forget to reward yourself for your efforts
Reminder	1 week after completing the last strategy	N/A	Reapplying a strategy can help you reach your goal
Reminder	3 days after the last strategy was completed	Only if strategy X was not started	Don't forget that there are other strategies you can apply
Reminder	1 day after strategy X was started	Only if strategy X was not completed	The last strategy you started is waiting for you!
