# Supplementary material for: Reducing Cannabis Use in Young Adults With Psychosis Using iCanChange, a Mobile Health App: Protocol for a Pilot Randomized Controlled Trial (ReCAP-iCC)
Source: JMIR Res Protoc. 2022 Nov 25;11(11):e40817. doi: 10.2196/40817 (PMC9736767; doi:10.2196/40817)
Supplement: Multimedia Appendix 2 [file resprot_v11i11e40817_app2.pdf]

Appendix 2. iCC Badges

**Section 1**
1. Introduction
**Engagement badge -** congratulate user for beginning the intervention
2. Cannabis dependence
3. Cannabis myths
4. Benefits of reducing
5. Believe in your strengths
6. Triggers
**Explorer badge** - for reflecting about personal reasons to change cannabis use and identifying their strengths
7. Withdrawal
8. Craving
9. Cannabis and psychosis
10. Choose a cannabis use goal
**Courage badge -** for having the determination to choose an objective related to cannabis use
**Section 2**
Supplementary module: Return to objective
**Objective met – If the participant reports achieving the cannabis consumption objective**
1. Doing cannabis-free activities
2. Getting social support
3. Taking care of yourself
4. Managing your stress
**Persistence badge*** - for their constant participation in the intervention and work towards achieving their objectives
5. Finding solutions to your problems
6. Communicating effectively
7. Being assertive
8. Valuing and rewarding yourself
**Skilled user badge** - for achieving new knowledge and skills related to reducing cannabis use
9. My journey (end of 12 weeks intervention)
**iCanChange champion** - for successfully completing the intervention

Note: ***** this badge will be unlocked after the fourth strategy explored but not specifically after “Manage my stress” because the strategies can be done in any order.

Badge name	Message
**Engagement**	Congratulations on completing the first module. The first step is always the hardest!
**Explorer**	Good work! You’ve thought about personal reasons for change and know your strengths. You can continue exploring!
**Courage**	You have chosen the goal that best suits you. This is a big step. Congratulations!
**Objective met**	Wow, you achieved your consumption goal! CONGRATULATIONS!<br>With your perseverance and ability to sustain efforts you will be able to achieve many other goals in your life; maybe another badge?
**Persistence**	Bravo, you are still with iCanChange. You are a persevering and curious person. You spend time and energy to reach your consumption goal. Great victories are won step by step. You’re on the right track.
**Skilled user**	Applause! You are now better equipped to meet your cannabis use goals.
**iCanChange Champion**	Bravo, you have maintained your focus and attention until the end, and you have completed the intervention. You are a champion iCanChange!

Note: If in the final module the participant reports that the cannabis consumption objective was achieved, two badges are unlocked: Objective met and iCanChange Champion
