# Supplementary figures and images for: Reducing Cannabis Use in Young Adults With Psychosis Using iCanChange, a Mobile Health App: Protocol for a Pilot Randomized Controlled Trial (ReCAP-iCC)
Source: JMIR Res Protoc. 2022 Nov 25;11(11):e40817. doi: 10.2196/40817 (PMC9736767; doi:10.2196/40817)

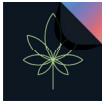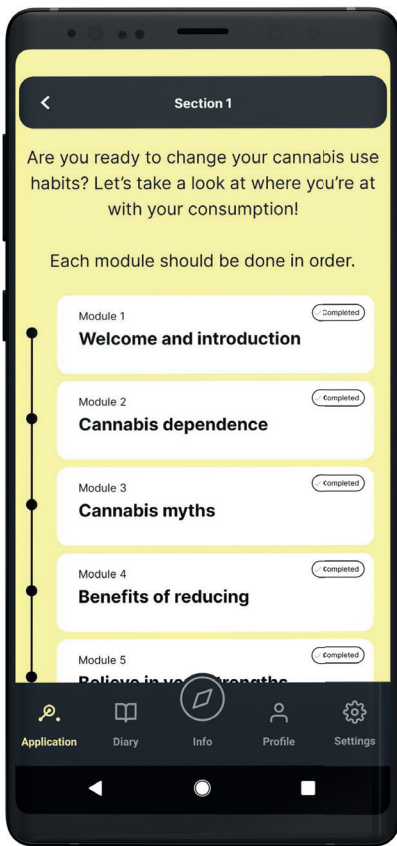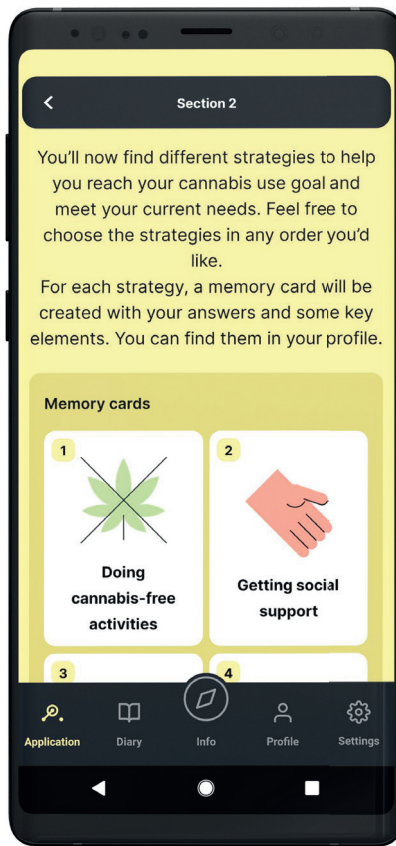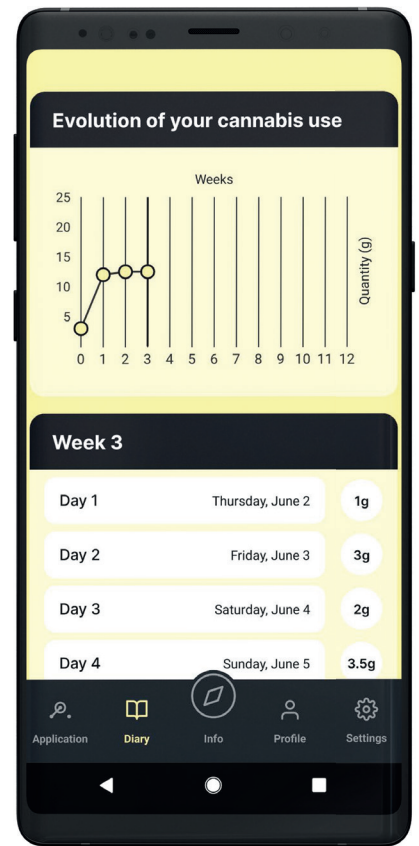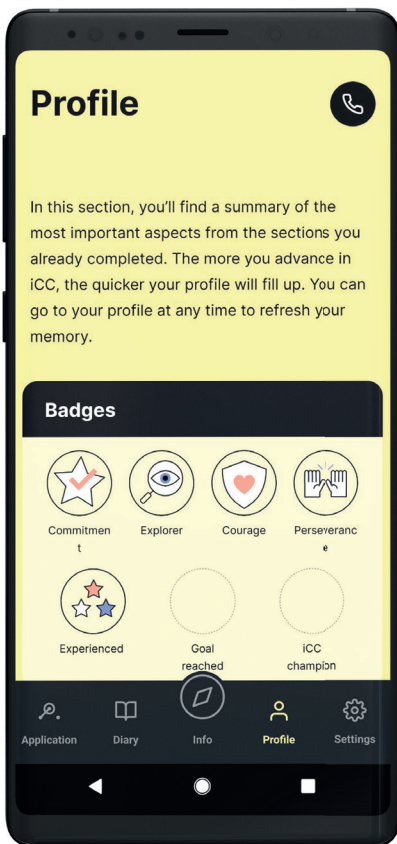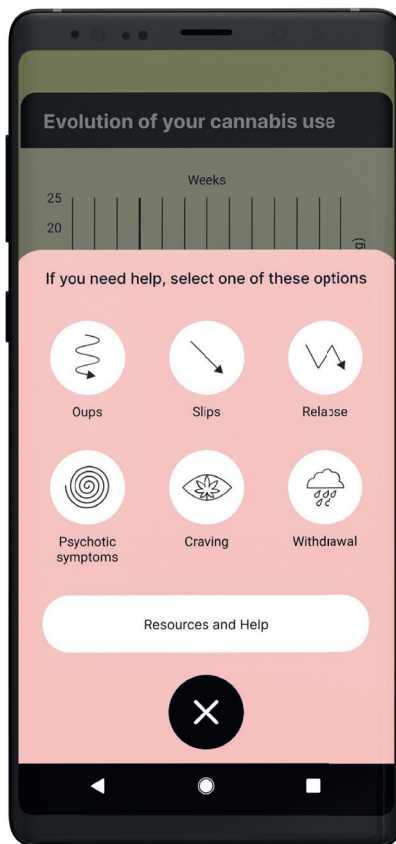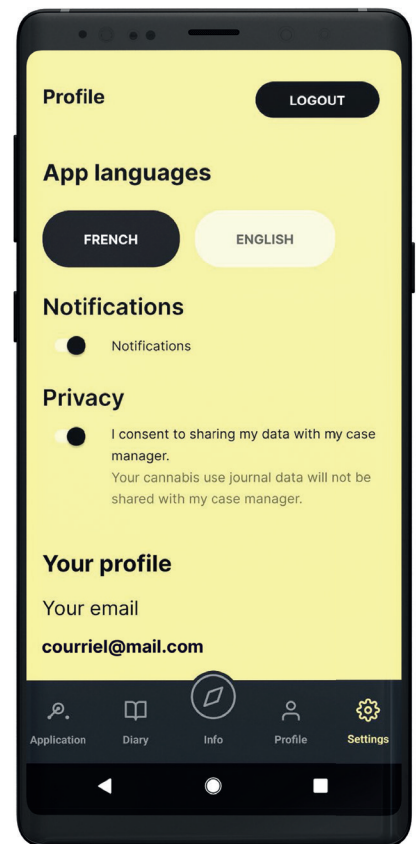

Supplement: Multimedia Appendix 3 [file resprot_v11i11e40817_app3.pdf]
